# Effects of Trace Elements on Anthropometric Characteristics of Children: Cobalt and Childhood Body Mass Index

**DOI:** 10.14789/jmj.JMJ21-0043-OA

**Published:** 2022-06-20

**Authors:** JUMPEI TETSUKA, TAKEHISA MATSUKAWA, KAZUHITO YOKOYAMA, SYUDO YAMASAKI, SHUNTARO ANDO, ATSUSHI NISHIDA, MARIKO HIRAIWA-HASEGAWA, KIYOTO KASAI

**Affiliations:** 1 Department of Epidemiology and Environmental Health, Juntendo University, Faculty of Medicine, Tokyo, Japan; 1 Department of Epidemiology and Environmental Health, Juntendo University, Faculty of Medicine, Tokyo, Japan; 2 Department of Forensic Science, Juntendo University, Faculty of Medicine, Tokyo, Japan; 2 Department of Forensic Science, Juntendo University, Faculty of Medicine, Tokyo, Japan; 3 Department of Epidemiology and Environmental Health, International University of Health and Welfare Graduate School of Public Health, Tokyo, Japan; 3 Department of Epidemiology and Environmental Health, International University of Health and Welfare Graduate School of Public Health, Tokyo, Japan; 4 Department of Psychiatry and Behavioral Science, Tokyo Metropolitan Institute of Medical Science, Tokyo, Japan; 4 Department of Psychiatry and Behavioral Science, Tokyo Metropolitan Institute of Medical Science, Tokyo, Japan; 5 Department of Neuropsychiatry, Graduate School of Medicine, The University of Tokyo, Tokyo, Japan; 5 Department of Neuropsychiatry, Graduate School of Medicine, The University of Tokyo, Tokyo, Japan; 6 Department of Evolutionary Studies of Biosystems, School of Advanced Sciences, The Graduate University for Advanced Studies (SOKENDAI), Kanagawa, Japan; 6 Department of Evolutionary Studies of Biosystems, School of Advanced Sciences, The Graduate University for Advanced Studies (SOKENDAI), Kanagawa, Japan; 7 International Research Center for Neurointelligence (WPI-IRCN), The University of Tokyo, Tokyo, Japan; 7 International Research Center for Neurointelligence (WPI-IRCN), The University of Tokyo, Tokyo, Japan

**Keywords:** cobalt, trace elements, gender, childhood obesity, body mass index

## Abstract

**Objectives:**

There are many reports on the effects of trace elements on human anthropometric characteristics. Among these elements, cobalt has consistently shown an inverse relationship with obesity risk. In the present study, we aimed to investigate the relationship between urinary levels of trace elements, focusing on cobalt, and childhood obesity, as indicated by the body mass index (BMI) in early adolescents, focusing on the participants' gender.

**Design:**

A cross-sectional study was conducted in the Tokyo Teen Cohort study. Based on urinary samples, we obtained the anthropometric characteristics (weight and height) and potential covariates associated with childhood BMI for 1542 children (mean age=12 years; 860 boys and 682 girls).

**Methods:**

Concentrations of urinary cobalt and 17 other trace elements were measured using inductively coupled plasma-mass spectrometry or inductively coupled plasma-atomic emission spectrometry.

**Results:**

Pearson's correlation coefficient revealed an inverse relationship between the log of cobalt concentrations in the urine and the BMI for the boys (r=-0.125, p<0.001) and girls (r=-0.082, p=0.033). Multivariate analysis, adjusted for various covariates, reconfirmed the correlation between urine cobalt and the childhood BMI, only in the boys (beta=-0.14, p<0.001).

**Conclusions:**

Among the 18 elements measured in the children's urine, cobalt may exhibit sufficient potency to decrease the risk of childhood obesity, particularly in boys. Future studies are required to clearly determine the magnitude of the effect and the underlying mechanism(s).

## Introduction

Over the past three decades, the frequency of childhood obesity has been increasing, leading to a worrisome epidemic worldwide^1)^. Currently, it is estimated that more than 38 million children under the age of 5 years and 340 million children/adolescents aged 5-19 years are overweight or obese^2)^. Owing to the risk of adulthood obesity^3)^, cardiometabolic mortality, and morbidity^4)^, childhood obesity is an important public health challenge^5, 6)^. Moreover, the Harvard Growth Study (1992) found that overweight adolescents had an increased risk of morbidity and mortality from coronary artery disease in the future, regardless of their adulthood weight^7)^.

Childhood obesity is a complex problem with a multifactorial etiology, including environmental, genetic, and ecological factors^8, 9)^. For example, excess calorie intake in children may be an intrinsic consequence of unhealthy eating habits. This may include insufficient intake of necessary nutrients or excessive consumption of toxic substances. Additionally, trace elements may contribute to obesity by influencing metabolism. Many previous studies have examined trace element exposure as a risk factor for childhood obesity, and have reported inverse associations between cobalt concentrations in various biological samples and obesity risk (BMI) in children^10-15)^ and adults^15-17)^. In addition, an experimental study demonstrated a difference in lipid profile (TG, HDL, and LDL) and body weight between mice exposed to cobalt and those that were not^18)^. However, such relationships between cobalt and glucose and lipid metabolism have not yet been revealed in humans^19)^.

Previous reports have suggested that the effects of trace metals may vary according to the child's gender, such as lowering of birth weight in male newborns due to elevation of arsenic or lead concentrations in maternal blood^20, 21)^ and increase in the body weight of adult female participants due to higher hair cadmium levels^22)^. Additionally, cobalt absorption and/or excretion can be influenced by gender, as serum and urine cobalt concentrations are higher in women than in men^23)^. Similarly, studies in France^24)^ and Taiwan^25)^ have reported higher urinary cobalt (UCo) concentrations in women than in men. Furthermore, regarding the gender differences in the lipid profile after the onset of puberty, it would be important to assess the relationship between cobalt and childhood obesity in early adolescent boys and girls.

In the present study, we aimed to measure 18 trace elements in urine samples from children to assess their relationship with childhood BMI, mostly focusing on cobalt. Because gender plays an important role in cobalt biokinetics and affects children's anthropometric characteristics, we additionally compared cobalt concentrations, BMI, and their correlations between boys and girls. To our best knowledge, this study is the first to investigate the relationship between UCo and childhood BMI in early adolescents, focusing on the participants' gender.

## Materials and methods

In the present retrospective, cross-sectional study, data and urinary samples were obtained from the Tokyo Teen Cohort (TTC)^26)^. The TTC is a birth cohort study conducted by the Tokyo Metropolitan Institute of Medical Science for investigating children's physiological and psychological development, including self-regulation and personalized values on adolescents and their primary parents (usually mothers). In this community- based survey, participants were recruited randomly from three municipalities in the Tokyo metropolitan area using the resident registry. Self-report questionnaires and interviews were conducted using 3171 children (10 years old at the baseline survey). In phase two of the study, the participants were aged 12 years when the data were collected for the current study. In this phase, urine samples were collected from 1582 children and stored at −80 °C until the metal analyses.

**Figure 1:**
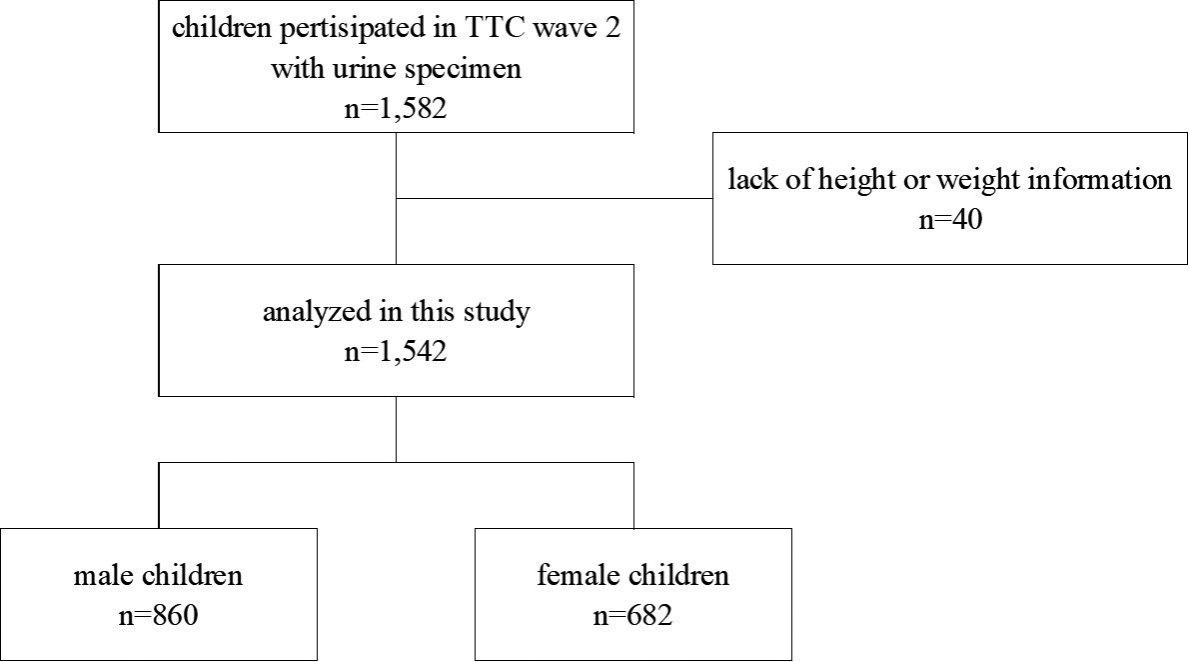
Study flow chart

We extracted data on body weight and height from the TTC dataset, as well as known potential covariates associated with childhood BMI, such as age (month), birth weight (g)^27)^, sleep duration (weighted average of weekday and weekend sleep hours per night)^28-30)^, parents' BMI^31-33)^, parental smoking^34)^, household income, and parents' education^35)^. Forty participants' data were excluded because of missing information regarding the children's height and/or weight. Finally, we included 1542 children, 860 boys (55.8 %) and 682 girls (44.2 %), for statistical analysis (Figure 1).

### Analysis of urine sample

Concentrations of trace elements in children's urine samples were measured by inductively coupled plasma-mass spectrometry (ICP-MS)^13, 36)^ or inductively coupled plasma-atomic emission spectrometry (ICP-AES), as previously reported^37)^. ICP-MS (Agilent 8800, Agilent Technologies, California, USA) was used for determining Li, V, Cr, Co, Ni, Cu, Zn, As, Se, Sr, Mo, Cd, Ba, and Tl, and ICP-AES (Optima 2100, PerkinElmer, Massachusetts, USA) was used for measuring Na, Ca, Mg, and K. For the measurement preparation, urine samples were melted at room temperature and mixed with 0.5 % HNO_3_ with 5-fold dilution in ICP-MS and analyzed by the multi-element standard solution XSTC-13 (SPEX CertiPrep, New Jersey, USA) as the external standard solution. For ICP-AES, the urine samples were diluted 10-fold with 0.5 % HNO_3_ and analyzed using XSTC-2A (SPEX CertiPrep, New Jersey, USA) as the external standard solution. Measurements were repeated three times, and the average of the three measurements was used for statistical analyses. For instrument calibration throughout the measurements, at least 10 % of the analyses were external standards, and 5 % were blank (pure water).

For statistical analysis, the values under the limit of detection (LOD) were substituted with half the LOD. To correct for variations in urine dilution, the concentration of every trace element was expressed as a ratio to urinary creatinine concentration. To reduce the influence of outliers and normalize the right-skewed distribution, we used the natural logarithm of the urinary concentration of trace elements in the statistical analysis. Among the 18 trace elements, we focused on cobalt, since several studies showed an inverse association of this element with childhood obesity (Table 1).

**Table 1:** Previous studies on relationship between trace elements, in different biological samples, and anthropometric characteristics

Country	Poland			USA	Turkey	Iran	Six countries from Europe	USA		USA	Russia
Age	6-17 y			6-19 y	Children with obesity: 10.59±2.90 y; healthy control: 10.71±2.07 y	20 mo to 3 y	6-11 y	Adolescents	Adults	All (women only)	All
Outcome	Obesity			Weight	BMI	Weight	BMI	BMI		BMI	Weight
Sample	Blood	Plasma	Urine	Urine	Serum	Hair	Urine	Urine		Toenails	Hair
Co	(−)	(−)	N/A	(−)	(−)	(−)	(−)	(−)	N/A	(−)	(−)
Li											N/A
Be											N/A
B											N/A
Mg										N/A	(−)
Al											(+)
Si											N/A
V					(−)					N/A	N/A
Cr										N/A	N/A
Mn	N/A	N/A	(+)							N/A	(−)
Fe	(−)	(−)	(−)							N/A	N/A
Ni	(+)	N/A	N/A							N/A	(−)
Cu	(−)	(−)	N/A				(+)			N/A	(−)
Zn	(−)	(−)	(−)			N/A				N/A	(−)
As										N/A	(+)
Se										N/A	N/A
Mo				(−)		N/A	(−)	N/A	N/A		
Cd	(+)	N/A	(+)	(−)	N/A	N/A		N/A	(−)	N/A	N/A
Sn										N/A	N/A
Sb				N/A		N/A		N/A	N/A	N/A	
I											(−)
Cs				N/A			(+)	N/A	(−)		
Ba				(+)	N/A			(+)	(+)		
W				N/A				N/A	N/A		
Hg										N/A	N/A
Tl				N/A				N/A	(+)		
Pb				(−)	N/A	N/A		(−)	(−)	N/A	N/A
Reference		10)		11)	12)	13)	14)	15)		16)	17)

(+): positive relationship, (−); negative relationship, N/A: no relationship

### Statistical analysis

Student's t-test (for continuous variables), Fisher's exact test (for categorical variables), or Mann- Whitney's U-test (ordinal scale) were used for comparisons between the two groups. Pearson's correlation coefficient was used to analyze the relationships between UCo and BMI. Multiple linear regression analysis was performed for assessing the relationships between UCo and urinary concentrations of the other 17 trace elements and BMI, controlling for possible confounding variables. All covariates were included using the forced-entry method. Analyses of all models were gender-stratified. The variance inflation factor (VIF) was employed for checking the multicollinearity problem among the variables. We used Bonferroni's correction to correct multiple comparisons, such as repeating the statistical tests 36 times (18 measured trace elements for both boys and girls), with a p- value<0.001 (0.05/36), which was considered to indicate a statistically significant difference. All statistical analyses were conducted using IBM Statistical Package for Social Sciences (SPSS), version 27.0 (IBM Corp., New York, USA).

## Results

The mean BMI of the children was 17.9 kg/m^2^ and very close between boys and girls (17.8 and 17.9 kg/m^2^, respectively). The mean log UCo was −0.295 μg/g, and there was no significant difference between boys and girls (mean=−0.308 and −0.279 μg/g, respectively). The Student's t-test showed a significant difference in mean birth weight between boys and girls (mean±SD=3062±412 and 2995±401 g, respectively, p=0.002). The statistical analysis did not indicate any significant differences in the other characteristics between boys and girls (Table 2).

**Table 2:** Comparison of continuous and categorical variables between boys and girls

Characteristics^a^	Total (n=1542)	Boys (n=860)	Girls (n=682)	p-value^b^
Log UCo	−0.295±0.317	−0.308±0.323	−0.279±0.308	0.075
Age (months)	145.6±3.6	145.6±3.5	145.5±3.7	0.745
Height (cm)	150.1±7.0	149.8±7.4	150.4±6.5	0.092
Weight (kg)	40.4±7.5	40.2±7.7	40.8±7.1	0.118
BMI (kg/m^2^)	17.9±2.4	17.8±2.4	17.9±2.4	0.243
Birthweight (g)	3032.2±408.2	3061.6±411.9	2995.2±400.8	0.002
Average sleep duration (hour)	8.8±0.7	8.8±0.7	8.8±0.7	0.357
Father's BMI	23.6±3.2	23.4±3.2	23.7±3.2	0.068
Mother's BMI	21.0±2.7	20.9±2.7	21.1±2.8	0.448
Parental smoking				0.821
Yes	460 (29.8)	256 (29.8)	204 (29.9)	
No	1006 (65.2)	552 (64.2)	454 (66.6)	
Unknown (%)	76 (4.9)	52 (6.0)	24 (3.5)	
Annual household income (10000 yen)				0.383
0-299	69 (4.5)	42 (4.9)	27 (4.0)	
300-599	349 (22.6)	205 (23.8)	144 (21.1)	
600-999	577 (37.4)	304 (35.3)	273 (40.0)	
1000+	528 (34.2)	295 (34.3)	233 (34.2)	
Unknown	19 (1.2)	14 (1.6)	5 (0.7)	
Father's education				0.811
High school or less	262 (17.0)	136 (15.8)	126 (18.5)	
2-year college	218 (14.1)	129 (15.0)	89 (13.0)	
4-year university	821 (53.2)	450 (52.3)	371 (54.4)	
Graduate university	176 (11.4)	98 (11.4)	78 (11.4)	
Unknown	65 (4.2)	47 (5.5)	18 (2.6)	
Mother's education				0.075
High school or less	248 (16.1)	140 (16.3)	108 (15.8)	
2-year college	687 (44.6)	402 (46.7)	285 (41.8)	
4-year university	544 (35.3)	284 (33.0)	260 (38.1)	
Graduate university	54 (3.5)	28 (3.3)	26 (3.8)	
Unknown	9 (0.6)	6 (0.7)	3 (0.4)	

^a^ Data presented as mean ± SD or number (percentage) ^b^ Comparison between boys and girls Student's t-test was used for continuous variables, Fisher's exact probability test was used for categorical variables, Mann-Whitney's U-test was used for ordinal scale

**Figure 2:**
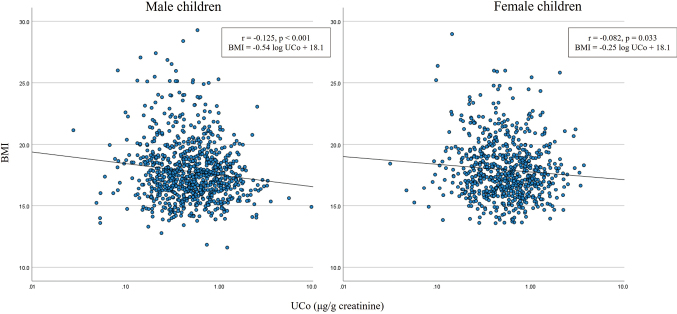
Pearson correlation coefficients between urinary cobalt concentration (UCo) and BMI in 860 boys and 682 girls

Pearson's correlation coefficient revealed a weak inverse correlation between log UCo and BMI in boys (r=−0.125, p<0.001) and girls (r=−0.082, p= 0.033) (Figure 2). Multiple linear regression analysis showed an inverse correlation between log UCo and BMI in boys after adjustment for confounding factors (beta=−0.14, p<0.001) (Table 3). The VIF did not demonstrate a multicollinearity problem among the predictor variables (VIF<2.0). In addition, the statistical analysis failed to indicate any significant relationship between the BMI and the urine levels of the other trace element (Table 4).

**Table 3:** Relationships of log UCo and other variables to BMI: Multiple linear regression analysis by the forced-entry method

	Boys (adjusted R^2^=0.137)			Girls (adjusted R^2^=0.096)	
	Beta^a^	p-value		Beta^a^	p-value
Log UCo	−0.14	<0.001		−0.06	0.106
Age (months)	0.06	0.075		0.10	0.011
Birthweight (g)	0.07	0.033		0.06	0.140
Average sleep duration (hours)	−0.14	<0.001		−0.05	0.185
Father's BMI	0.18	<0.001		0.14	<0.001
Mother's BMI	0.19	<0.001		0.20	<0.001
Parental smoking (Yes=1, No=0)	0.09	0.013		0.02	0.586
Father's education (years)	0.02	0.685		−0.07	0.094
Mother's education (years)	0.04	0.291		0.04	0.350
Annual household income	0.01	0.784		0.07	0.122

^a^ Standardized partial regression coefficien

**Table 4:** Relationships of 18 trace elements to BMI

Log urinary concentration^a^	Boys			Girls	
	Beta^b^	p-value		Beta^b^	p-value
Li	−0.08	0.029		−0.04	0.328
Na	−0.06	0.093		−0.00	0.910
Mg	−0.08	0.020		−0.06	0.138
K	−0.07	0.061		0.01	0.848
Ca	−0.02	0.501		0.08	0.046
V	−0.03	0.348		−0.03	0.418
Cr	−0.02	0.495		−0.03	0.525
Co	−0.14	<0.001		−0.06	0.106
Ni	−0.08	0.019		−0.07	0.095
Cu	−0.06	0.079		−0.06	0.126
Zn	−0.06	0.079		−0.08	0.051
As	−0.05	0.126		0.00	0.926
Se	0.02	0.501		0.05	0.233
Sr	−0.04	0.227		0.02	0.536
Mo	−0.05	0.133		−0.09	0.018
Cd	−0.02	0.649		−0.05	0.208
Ba	−0.05	0.170		−0.01	0.785
Tl	−0.03	0.453		0.01	0.900

^a^ Adjusted for age, birthweight, average sleep duration, father's BMI, mother's BMI, parental smoking, annual household income, father's education, and mother's education, by multiple linear regression analysis using the forced-entry method ^b^ Standardized partial regression coefficient

## Discussion

This study's findings showed that increasing UCo levels were associated with a decrease in BMI in boys. This association was confirmed after adjustment for several covariates, including genetic, behavioral, and environmental factors, in the multivariate analysis. Similarly, many previous studies have shown an inverse association between cobalt and obesity/overweight rates, regardless of age, gender, and type of the biological samples. Among them, five studies assessed only children^10-14)^, one study had no age limitation^15)^, and two studies assessed adults^16, 17)^.

Consistent with the present study, Padilla (2010)^15)^, Shao (2017)^11)^, and Vrijheid (2020)^14)^ reported an inverse association between UCo levels and childhood BMI and weight. Błażewicz (2013)^10)^ showed lower plasma and blood cobalt concentrations in children with obesity than in those without obesity; however, the study failed to demonstrate the same results for UCo. Similarly, Vigeh (2017) and Skalnaya (2018) reported higher levels of hair cobalt in children and adults with low body weight than in those with normal weight^13, 17)^. In adult women and children, an inverse correlation has been reported between cobalt levels in the toenail^16)^ and serum^12)^, respectively, and BMI. In addition, experimental studies (mice and rats) have shown that blood/urine/serum cobalt produce the same effects on animal weight/BMI^18, 38)^. Thus, the findings of the current study and previous epidemiological/experimental studies suggest that cobalt may reduce the risk of obesity.

Although many previous studies ignored the bioavailability of trace elements and their effects according to the subjects' gender, the present study examined UCo levels and stratified the effects by the participants' gender. There was no significant difference in the UCo levels between boys and girls in the present study. However, previous studies have reported higher levels of UCo in women than in men^23-25)^. This difference may be induced by the greater iron demand in women. Cobalt and iron may share a common intestinal uptake mechanism^39)^; thus, iron deficiency (a common problem in young women) increases cobalt absorption and urinary excretion in animals^40)^ and humans^41)^. Since the participants of the present study were in early adolescence (56 % of the girls did not experience menarche), the difference in UCo between girls and boys could not be detected at their ages.

Pearson's correlation analysis revealed an inverse correlation between log UCo and BMI in both boys and girls. When we adjusted for confounding factors, in the multiple linear regression analysis, a significant correlation was demonstrated only in the boys. These gender-related findings suggest that UCo is a protective factor against childhood obesity, predominantly in boys. The molecular or biochemical mechanisms underlying the reduction of BMI by cobalt have not been clearly understood. Tascilar (2011) found a correlation between plasma cobalt and the insulin resistance index (HOMA-IR), which suggests that cobalt acts as a regulator of glycogen depot by suppressing glucagon signaling and its effect on body weight^12)^. In rats, cobalt administration was reported to result in decreased blood glucose levels, regulated glucose tolerance, and reduced body weight^38)^. In addition, cobalt can decrease obesity risk by altering lipid metabolism, such as increasing leptin; the magnitude of the effect varies according to gender. For instance, leptin levels in women are higher after the onset of puberty^42, 43)^. Similarly, cobalt increases plasma HDL cholesterol and decreases LDL cholesterol, free fatty acids, and triglycerides^18)^. Although cobalt may reduce childhood BMI by influencing lipid metabolism, we did not determine the level of leptin in the participants of the present study. Another possible underlying mechanism might be iron metabolism, as iron plays an important role during rapid growth periods, such as adolescence. A recent study reported lower iron concentrations in children and adolescents who are overweight and a 50 % incidence of iron-deficiency anemia in individuals with a BMI above the 97th percentile^44)^. Cobalt may influence iron metabolism, consequently increasing obesity risk by increasing the hemoglobin, hematocrit, and red blood cell counts in men^45)^. Thus, cobalt may influence BMI by changing the metabolism of glucose, lipids, and iron differently in men and women.

In the present study, with a relatively large sample size, we considered several potentially confounding factors. However, the limitations of this study need to be addressed. First, the cross-sectional design of the present study may not draw conclusions regarding the causal relationship between UCo and childhood BMI. The generalizability of the results might be limited owing to the study design. Second, child anthropometric characteristics (i.e., weight and height) develop over several months to years; thus, a single UCo measurement may not reflect cumulative concentrations or exposure levels at an earlier life. Third, urinary excretion of cobalt is multiphasic, with a rapid increase in hours and a peak of elimination at 24 h following exposure^46)^. In other words, the current study obtained the level of cobalt exposure via measurement of one spot-urine sample. It would be better to collect a 24 h urine sample for cobalt measurements. Finally, some considerable confounding factors associated with weight and BMI, such as the participants' diet and physical activities, were not adjusted for in the current study because information on these was limited in the TTC dataset.

In summary, among the 18 measured trace elements in the present study, only cobalt showed a significant inverse relationship with BMI in Japanese boys. Thus, cobalt may have sufficient potency to decrease the risk of obesity in children. Future epidemiological and experimental studies may need to clarify the magnitude of the effect and underlying mechanism(s).

## Funding

This work was supported by a Grant-in-Aid for Scientific Research on Innovative Areas (23118002; Adolescent Mind & Self-Regulation) from the Ministry of Education, Culture, Sports, Science and Technology of Japan and Japan Society for the Promotion of Science (JSPS) KAKENHI (Grant Number JP19H01081).

## Author contributions

Conception and design, JT and TM. Formal analysis and drafting of the article, JT. Critical revision for important intellectual content, KY, SY, SA, AN, MHH, and KK. Measurement of trace elements' concentration, TM. All authors read and approved the final manuscript.

## Conflicts of interest statement

The authors declare that they have no conflicts of interest.

## Ethics approval

The present study was conducted after approval was received from the ethics committees of the Tokyo Metropolitan Institute of Medical Science (approval no. 14-08) and Juntendo University (approval no. 2016092).

## Consent to participate

Written informed consent was obtained from each participant and the participant's primary parent before participation, as part of the Tokyo Teen Cohort study.
